# Neoplasias e a Avaliação do Risco de Doença Cardiovascular

**DOI:** 10.36660/abc.20220007

**Published:** 2022-02-14

**Authors:** Cristina Salvadori Bittar

**Affiliations:** 1 InCor São Paulo SP Brasil Instituto do Coração - InCor,São Paulo, SP – Brasil; 2 ICESP São Paulo SP Brasil Instituto do Câncer - ICESP,São Paulo, SP – Brasil; 3 Hospital Sírio Libanês São Paulo SP Brasil Hospital Sírio Libanês, São Paulo, SP – Brasil

**Keywords:** Doença da Artéria Coronariana/complicações, Neoplasias Pulmonares/complicações, Angiografia Coronária Percutânea/métodos, Índice de Gravidade de Doença, Intervenção Coronária Percutânea/métodos

A doença cardiovascular (DCV) e as neoplasias são, na atualidade, as duas principais causas de mortalidade no Brasil e no mundo.^1^ Tanto a incidência de câncer quanto a de doença cardiovascular aumentam com a idade, sendo que 77% dos casos de câncer são diagnosticados em pacientes com mais de 55 anos e, concomitantemente, o risco cardiovascular também é maior em pacientes homens acima de 55 anos e mulheres acima de 65 anos. No Brasil, em 2016, as DCV apresentaram as maiores taxas de mortalidade e anos de vida perdidos ajustados por incapacidade, em ambos os sexos.^2^

Devido ao grande impacto das duas doenças mais prevalentes que emergem em paralelo e interagem entre si, é importante considerarmos como manejar essas duas doenças individualmente e do ponto de vista populacional. Além disso, poucos estudos exploraram a população de indivíduos com uma “carga dupla” dessas doenças, conhecida como “ *double burden* ”.^3^

Essas patologias compartilham uma série de fatores de risco modificáveis e mecanismos fisiopatológicos que frequentemente coexistem nos mesmos pacientes. ^4^

O câncer de pulmão é a causa número 1 de morte por câncer em homens e a causa número 2 em mulheres em todo o mundo. Evidências de vários estudos apoiam uma forte associação entre câncer de pulmão e DCV: os pacientes com câncer de pulmão têm um risco aumentado de aproximadamente 66% de DCV e um risco aumentado de 89% de doença arterial coronariana isoladamente em comparação com aqueles sem câncer de pulmão.^5^

No estudo realizado por Mingzhuang Sun et al.^6^ publicado neste volume do Arquivos Brasileiros de Cardiologia, foi realizada uma análise transversal de 75 pacientes recém-diagnosticados com neoplasia de pulmão, entre 2009 e 2019, em um hospital em Beijing, China (Chinese PLA General Hospital). Esses pacientes foram submetidos a cineangiocoronariografia e comparados com 225 pacientes-controle sem neoplasia.

Os pacientes com tratamento cardiológico (angioplastia ou revascularização coronariana) ou com tratamento oncológico prévio foram excluídos.

A gravidade da doença coronariana foi avaliada utilizando o escore SYNTAX, sendo dividido em quartis, com base na distribuição do escore em todos os indivíduos do estudo.

A maioria dos pacientes com neoplasia foi diagnosticada com o tipo não pequenas células (92%) em estágio I e II (75%). Em pacientes com câncer de pulmão, o escore SYNTAX foi de 20,0%, 20,0%, 24,0% e 36,0% nos pacientes nos quartis mais baixo, médio-inferior, médio-superior e mais alto, respectivamente. Nos pacientes-controle, a porcentagem de pacientes foi de 26,7%, 26,2%, 25,8% e 21,3% do quartil mais baixo ao mais alto, respectivamente. Houve uma diferença na distribuição dos escores entre os pacientes com câncer de pulmão e os pacientes-controle, tendo o grupo com câncer uma porcentagem significativamente mais alta de pacientes com escores SYNTAX mais alto (36% versus 21,6%).

A mensagem principal que o estudo levanta é a de que existem evidências epidemiológicas mostradas neste e em outros estudos sobre a relação existente entre o diagnóstico oncológico, principalmente nos casos de câncer de pulmão, mama e cólon e o risco aumentado de DCV.^7^ Dessa forma, o diagnóstico dessas neoplasias deve desencadear a lembrança de uma avaliação de risco de DCV concomitante, uma vez que o tratamento e, consequentemente, o prognóstico de ambas as patologias evoluiu grandemente nos últimos anos, com aumento de sobrevida.^8^ Sendo assim, trata-se de tema importante tanto para os cardio-oncologistas quanto para os cardiologistas em geral, dada a alta prevalência. Neste estudo, a avaliação coronariana foi realizada através de cineangiocoronariografia, mas levanta também uma reflexão e algumas hipóteses sobre o melhor método de avaliação de DCV nesta população, dadas as particularidades e a complexidade dos pacientes nesta situação.

Trata-se de um estudo realizado na população chinesa que nos lembra da necessidade de geração e busca de dados nacionais para conhecimento do nosso perfil populacional e planejamento de ações futuras de doenças tão prevalentes.

## References

[1] Hajjar LA, Costa I, Lopes M, Hoff PMG, Diz M, Fonseca SMR, et al. Brazilian Cardio-oncology Guideline - 2020. Arq Bras Cardiol. 2020;115(5):1006-43. doi: 10.36660/abc.20201006 10.36660/abc.20201006 PMC8452206 33295473

[2] Malta DC, Pinheiro PC, Teixeira RA, Machado IE, Santos FMD, Ribeiro ALP. Cardiovascular Risk Estimates in Ten Years in the Brazilian Population, a Population-Based Study. Arq Bras Cardiol. 2021;116(3):423-31. doi: 10.36660/abc.20190861 10.36660/abc.20190861 PMC8159568 33909770

[3] Kreatsoulas C, Anand SS, Subramanian SV. An emerging double burden of disease: the prevalence of individuals with cardiovascular disease and cancer. J Intern Med. 2014;275(5):494-505. doi: 10.1111/joim.12165 10.1111/joim.12165 24372982

[4] Koene RJ, Prizment AE, Blaes A, Konety SH. Shared Risk Factors in Cardiovascular Disease and Cancer. Circulation. 2016;133(11):1104-14. doi: 10.1161/CIRCULATIONAHA.115.020406 10.1161/CIRCULATIONAHA.115.020406 PMC4800750 26976915

[5] Yuan M, Li QG. Lung Cancer and Risk of Cardiovascular Disease: A Meta-analysis of Cohort Studies. J Cardiothorac Vasc Anesth. 2018;32(1):e25-e27. doi: 10.1053/j.jvca.2017.04.033. 10.1053/j.jvca.2017.04.033 29150236

[6] Sun M, Yang Q, Li M, Jing J, Zhou H, Chen Y, et al. Associação entre a gravidade da doença arterial coronariana e cancer do pulmão: um estudo piloto transversal. Arq Bras Cardiol. 2022; 118(2):478-485. 10.36660/abc.20200478 PMC8856686 35262584

[7] Handy CE, Quispe R, Pinto X, Blaha MJ, Blumenthal RS, Michos ED, et al. Synergistic Opportunities in the Interplay Between Cancer Screening and Cardiovascular Disease Risk Assessment: Together We Are Stronger. Circulation. 2018;138(7):727-34. doi: 10.1161/CIRCULATIONAHA.118.035516 10.1161/CIRCULATIONAHA.118.035516 30359131

[8] Kravchenko J, Berry M, Arbeev K, Lyerly HK, Yashin A, Akushevich I. Cardiovascular comorbidities and survival of lung cancer patients: Medicare data based analysis. Lung Cancer. 2015;88(1):85-93. doi: 10.1016/j.lungcan.2015.01.006. 10.1016/j.lungcan.2015.01.006 PMC4375130 25704956

