# Supplementary material for: Neoplasias e a Avaliação do Risco de Doença Cardiovascular
Source: Arq Bras Cardiol. 2022 Feb 14;118(2):486–7. [Article in Portuguese] doi: 10.36660/abc.20220007 (PMC8856679; doi:10.36660/abc.20220007)
Supplement: Translation

# Translation: Neoplasms and the Evaluation of Risk of Cardiovascular Disease

**Authors:** Cristina Salvadori Bittar

**Affiliations:** 1 Brazil Instituto do Coração - InCor, São Paulo, SP – Brazil; 2 Brazil Instituto do Câncer - ICESP, São Paulo, SP – Brazil; 3 Brazil Hospital Sírio Libanês, São Paulo, SP – Brazil

**Keywords:** Coronary Artery Disease/complications, Lung Neoplasms/complications, Coronary Angiography/methods, Severity of Illness Index, Percutaneous Coronary Intervention/methods

Cardiovascular disease (CVD) and neoplasms are currently the two leading causes of mortality in Brazil and in the world.^1^ Both the incidence of cancer and neoplasms increase with age, with 77% of cancer cases being diagnosed in patients older than 55 and, concomitantly, the cardiovascular risk is also higher in male patients older than 55 and women older than 65. In Brazil, in 2016, CVDs had the highest rates of mortality and disability-adjusted years of life lost, in both sexes.^2^

Due to the great impact of the two most prevalent diseases that emerge in parallel and interact with each other, it is important to consider how to manage these two diseases individually and from a population point of view. In addition, few studies have explored the population of individuals with a double burden of these diseases.^3^

These pathologies share a number of modifiable risk factors and pathophysiological mechanisms that often coexist in the same patients.^4^

Lung cancer is the number 1 cause of cancer death in men and the number 2 cause of cancer death in women worldwide. Evidence from several studies supports a strong association between lung cancer and CVDs: patients with lung cancer have an approximately 66% increased risk of CVD and an 89% increased risk of coronary artery disease alone compared with those without lung cancer.^5^

In the study conducted by Mingzhuang et al.^6^ published in this volume of Arquivos Brasileiros de Cardiologia, a cross-sectional analysis was performed of 75 newly diagnosed patients with lung cancer between 2009 and 2019 at a hospital in Beijing, China (Chinese PLA General Hospital). These patients underwent coronary angiography and were compared with 225 control patients without neoplasms.

Patients under cardiological treatment (angioplasty or coronary artery bypass grafting) or with previous cancer treatment were excluded.

The severity of coronary artery disease was assessed using the SYNTAX score, divided into quartiles, based on the distribution of the score across all study subjects.

Most patients with neoplasms were diagnosed with the non-small cell type (92%) in stage I and II (75%). In lung cancer patients, the SYNTAX score was 20.0%, 20.0%, 24.0%, and 36.0% in the patients in the lowest, lowest-middle, upper-middle, and highest quartiles, respectively. In control patients, the percentage of patients was 26.7%, 26.2%, 25.8% and 21.3% from the lowest to the highest quartile, respectively. There was a trend in the distribution of patient scores that differed between lung cancer patients and control patients, with the cancer group having a significantly higher percentage of patients with higher SYNTAX scores (36% versus 21.6%).

The main message that the study conveys is that there is epidemiological evidence shown in this and in other studies about the relationship between oncological diagnosis, especially in cases of lung, breast and colon cancer, and the increased risk of CVD.^7^ Therefore, diagnosis of these neoplasms should trigger the reminder of a risk assessment of concomitant CVD, since the treatment and, consequently, the prognosis of both pathologies has greatly evolved in recent years, with increased survival.^8^ It is then an important topic for both cardio-oncologists and cardiologists in general, given its high prevalence. In this study, coronary evaluation was performed using coronary angiography, but it also raises a reflection and some hypotheses about the best method for evaluating CVDs in this population, given the particularities and complexity of patients in this situation.

This is a study carried out with the Chinese population that reminds us of the need to generate and search for national data in order to have better knowledge about our population profile and plan future actions for such prevalent diseases.
